# Macrophage pyroptosis in inflammatory bowel disease: mechanistic insights, pathological roles and treatment strategies

**DOI:** 10.3389/fimmu.2026.1904376

**Published:** 2026-09-08

**Authors:** Zhouxin Yu, Zhiqiang Zhao, Yushi Tian, Gengchen Xue, Jingbo Wang, Yuncong Li, Heng Fan

**Affiliations:** Department of Integrated Traditional Chinese and Western Medicine, Union Hospital, Tongji Medical College, Huazhong University of Science and Technology, Wuhan, China

**Keywords:** inflammasome, inflammatory bowel disease, macrophage, pyroptosis, therapeutic targeting

## Abstract

Inflammatory bowel disease (IBD) is a chronic, relapsing inflammatory disorder of the intestinal tract. Imbalanced intestinal immune homeostasis, particularly macrophage pyroptosis, plays a central role in the pathogenesis of IBD. Pyroptosis is a pro-inflammatory form of programmed cell death mediated by the gasdermin protein family. It is characterized by cellular swelling, membrane rupture, and the massive release of inflammatory mediators. Under physiological conditions, moderate pyroptosis contributes to the maintenance of intestinal immune homeostasis by eliminating intracellular pathogens and releasing immunomodulatory signals. However, aberrant activation of pyroptosis triggers the excessive release of inflammatory mediators and damage signals that drive the initiation and progression of intestinal inflammation. Recent studies have shown that macrophage pyroptosis is profoundly involved in the pathological evolution of IBD. Upon stimulation by danger signals, macrophages undergo pyroptosis and release a high amount of inflammatory mediators that remodel the local immune network. Macrophage pyroptosis can also directly impair the intestinal mucosal barrier, thereby driving the pathological progression of IBD. This article systematically reviews the molecular mechanisms involved in pyroptosis, summarizes the pathological mechanisms through which macrophage pyroptosis drives IBD progression, and introduces various therapeutic strategies targeting macrophage pyroptosis to alleviate IBD. Finally, this review provides novel perspectives for the mechanistic research and precision treatment of IBD and facilitates the clinical translation of strategies targeting macrophage pyroptosis.

## 1 Introduction

IBD, which is divided into Crohn’s disease (CD) and ulcerative colitis (UC), is a group of chronic, idiopathic inflammatory disorders (1). UC presents with a diffuse, continuous, and superficial inflammation localized to the colon, which typically ascends proximally from the rectum and is strictly confined to the mucosal layer (2). In contrast, CD can involve any region of the gastrointestinal tract and is characterized by focal, transmural inflammation often complicated by fistulas, abscesses, and stricture formation (3). The incidence of IBD has increased in recent years. Early industrialized regions have entered the compounding prevalence stage, where the prevalence of IBD has stabilized at a high level of 0.5%-1.0%. Conversely, newly industrialized countries in Asia are currently experiencing an acceleration phase in terms of incidence and are projected to transition into the compounding prevalence stage within the next two decades (4, 5). Therefore, IBD imposes a substantial medical and economic burden. IBD develops as a result of a combination of genetic susceptibility, environmental triggers, abnormal immunological responses, and an imbalance in the gut microbiota (6–9). Currently, the therapeutic armamentarium for IBD predominantly consists of aminosalicylates, glucocorticoids, immunosuppressants, and biologic agents. Nevertheless, a subset of patients does not respond to conventional treatments, and prolonged pharmacological intervention is often associated with an increased risk of opportunistic infections. This underscores a pressing need for novel targeted therapeutic strategies (10).

Disruption of intestinal immune homeostasis accelerates IBD progression. This disruption results largely from aberrant innate immune system activation (11). Macrophages, representing a highly plastic subset of innate immune cells, are fundamentally responsible for preserving intestinal immune tolerance under physiological steady-state conditions (12). Conversely, these intestinal macrophages undergo profound phenotypic shifts in IBD. They polarize into a pro-inflammatory M1 state, secrete abundant inflammatory cytokines, and engage in metabolic reprogramming. Together, these events synergistically perpetuate inflammation and tissue destruction (13, 14).

Following recent breakthroughs in programmed cell death, pathogenic macrophages extend far beyond the mere secretion of inflammatory cytokines and potentiation of the inflammatory cascade. Crucially, the stimulus-driven shift in their intrinsic cell death modalities profoundly orchestrates disease progression (15). Upon exposure to pathogen-associated molecular patterns (PAMPs) and damage-associated molecular patterns (DAMPs), intestinal macrophages do not follow the typical apoptosis-driven physiological death pathway. Instead, they go through pyroptosis, a robust, pro-inflammatory type of cell death. Pyroptosis features cellular swelling, lytic membrane rupture, and massive efflux of inflammatory mediators, and is fundamentally dependent on the gasdermin (GSDM) family. Intrinsically, pyroptosis is essential for both immune surveillance of abnormal cells and host defense against invasive microorganisms. However, it is closely linked to the onset of IBD when it is overactivated (16).

Emerging as a critical pathological driver of intestinal inflammation, macrophage pyroptosis has recently garnered extensive attention, as its aberrant activation is strongly correlated with the severity of IBD in clinical settings (15). The massive release of inflammatory mediators by pyroptotic macrophages orchestrates the recruitment of immune cells and actively disrupts the integrity of the intestinal mucosal barrier. This establishes a detrimental “pyroptosis-inflammation” positive feedback loop that relentlessly contributes to disease progression. Although it is increasingly recognized that macrophage pyroptosis contributes to the pathogenesis of IBD, a dedicated review addressing this specific paradigm is currently lacking. To bridge this gap, this review comprehensively delineated the molecular basis of pyroptosis, elucidated the contribution of macrophage pyroptosis to the development of IBD, and systematically summarized current therapeutic strategies targeting this pathway. By outlining future research trajectories, this study offers novel theoretical insights to advance mechanistic research and targeted therapy in IBD.

## 2 Intestinal macrophages

Intestinal macrophages are essential for maintaining gut immune homeostasis. Their unique ontogeny and renewal kinetics give them profound heterogeneity and environmental plasticity. These properties allow them to maintain immune tolerance to commensal microbiota, while having an effective defensive response to invading pathogens (12, 17). This homeostatic state is essential for intestinal tissue. Its dysregulation drives the pathogenesis of IBD and tissue damage.

### 2.1 Ontogeny and heterogeneity of intestinal macrophages

The ontogeny and renewal kinetics of intestinal macrophages exhibit remarkable and unique features. Brain microglia originate from the yolk sac. Following early embryonic colonization, they establish a relatively isolated “immune-privileged” environment and rarely rely on peripheral blood replenishment throughout human life (18). Kupffer cells in the liver primarily stem from the fetal liver. They show a certain degree of monocytic input around birth, but their population is maintained predominantly through local proliferation in adulthood (19). Intestinal macrophages also reveal precursor cell colonization during embryogenesis. In adulthood, a fraction of intestinal macrophages maintains itself locally and independently, whereas the remainder relies on the recruitment and replenishment of peripheral monocytes (20). At the neurovascular-immune interface, there exists a specific subset of macrophages originating from both embryonic precursors and adult bone marrow-derived monocytes. Upon colonization, these macrophages can survive for a long period and self-renew, which is essential for the maintenance of intestinal vascular integrity and neurological function (21). For the more broadly distributed macrophages in the gut, their ontogeny and renewal highly rely on the “monocyte waterfall” paradigm (22). Specifically, peripheral blood-derived Ly6C^+^ monocytes are recruited to the intestinal lamina propria via a C-C chemokine receptor type 2-dependent pathway (23). Local microenvironmental signals induce a continuous and irreversible phenotypic evolution in monocytes (24). As differentiation progresses, these cells gradually downregulate the expression of the pro-inflammatory marker Ly6C, while simultaneously overexpressing major histocompatibility complex II (MHCII) and chemokine (C-X3-C motif) ligand 1 (CX3CR1) (25). Ultimately, these cells develop into Ly6C^-^MHCII^+^CX3CR1^+^ mature tissue-resident macrophages characterized by profound immune tolerance. Intestinal inflammation and pathological damage occur once this homeostatic state is disrupted (26, 27).

**Figure 1:**
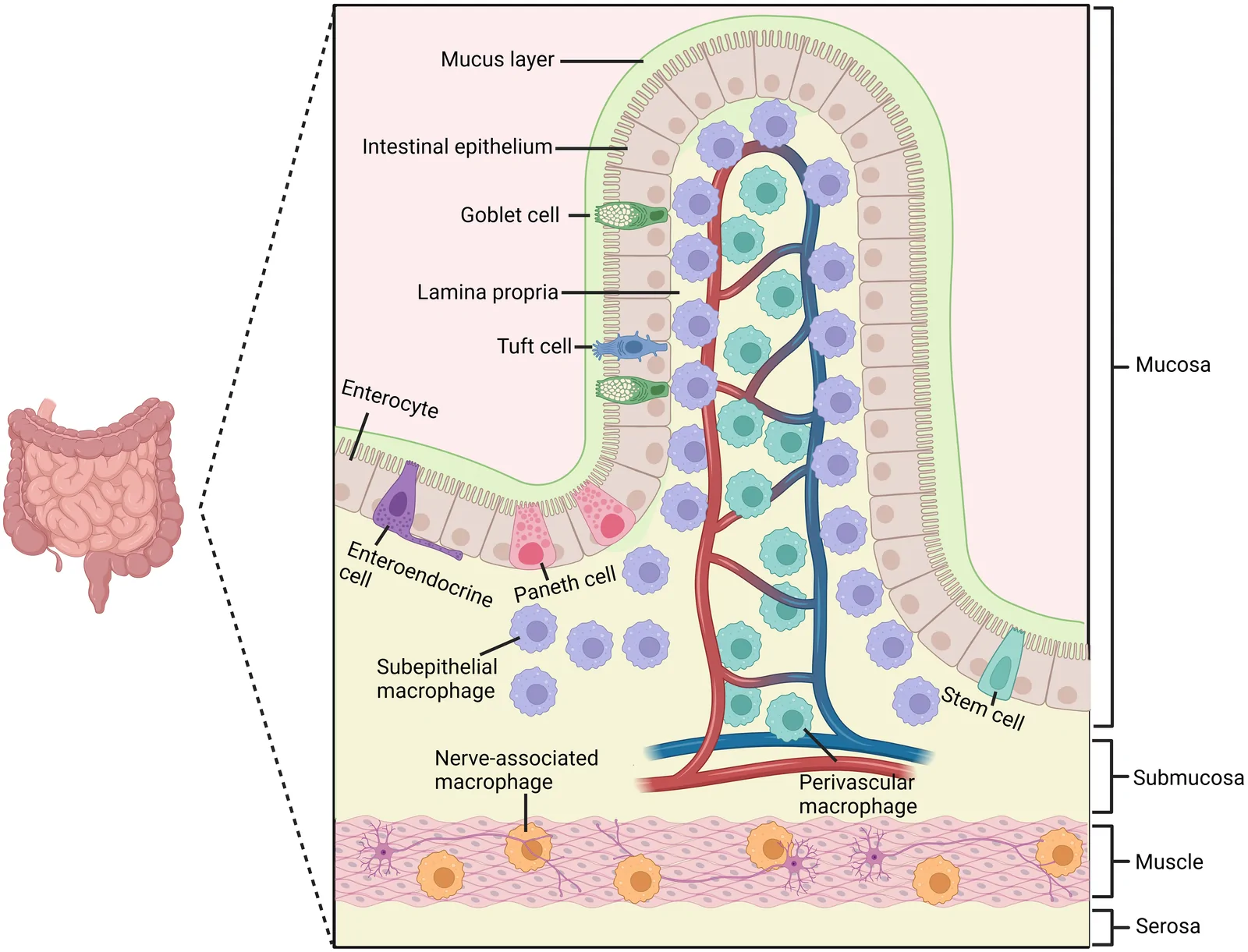
Spatial distribution characteristics of intestinal macrophages. Based on distinct spatial niches, intestinal macrophages are primarily classified into three subpopulations. Subepithelial macrophages, located in the upper lamina propria beneath the intestinal epithelium, are responsible for immune surveillance and maintaining immune tolerance. Perivascular macrophages adhere to submucosal blood vessels, monitoring microvascular permeability. Nerve-associated macrophages reside in the muscularis layer, participating in the maintenance of the enteric nervous system and intestinal peristalsis.

Intestinal macrophages are highly heterogeneous, with their functional identities dictated by specific spatial niches. Based on these spatial niches, intestinal macrophages can be classified into subepithelial macrophages, perivascular macrophages, and nerve-associated macrophages (28). Subepithelial macrophages constitute the vast majority of intestinal macrophages. Located beneath the intestinal epithelial barrier, they exhibit a rapid turnover rate and highly depend on continuous replenishment by peripheral blood monocytes (23). In physiological conditions, subepithelial macrophages display a typical state of inflammatory anergy. While possessing robust phagocytic and bactericidal capacities, they exhibit profound tolerance to microbial antigens. By continuously secreting factors, they promote the differentiation of regulatory T cells (Treg), thereby clearing pathogens while maintaining immune tolerance (29). Perivascular macrophages are distributed in the perivascular space within the submucosa. They are responsible for monitoring submucosal microvascular permeability and preventing the systemic distribution of pathogens that have breached the mucosal layer (21, 30). Nerve-associated macrophages closely interact with the enteric nervous system and smooth muscle cells. They serve as a critical hub for maintaining intestinal motility (31) (**Figure 1**).

### 2.2 Pathogenic remodeling of intestinal macrophages in IBD

The immune homeostasis of the intestinal microenvironment is disrupted in the development and progression of IBD, accompanied by an increased abundance of macrophages and profound phenotypic shifts (28). In the presence of chemokines released from damaged tissues, the massive recruitment of peripheral blood monocytes to inflammatory sites, where they differentiate into macrophages, constitutes the primary cause of macrophage accumulation in the inflamed gut (20). Newly differentiated macrophages are initially in an M0 state and exhibit phagocytic and immune surveillance functions (32). These macrophages show considerable phenotypic plasticity and can differentiate into either a pro-inflammatory M1 or an anti-inflammatory M2 phenotype, depending largely on microenvironmental signals. Under homeostatic conditions, macrophages display an M2-like anti-inflammatory phenotype that supports the function of Treg cells and prevents excessive immune activation by releasing cytokines, such as interleukin-10 (33). Furthermore, M2 macrophages maintain the production of tight junction (TJ) proteins and promote the secretion of mucin 2 (MUC2). These macrophages also promote epithelial regeneration (34–36). In this state, the pro-inflammatory M1 phenotype is maintained at low levels, such that a finely tuned dynamic equilibrium exists between the two populations. In contrast, the pathogenic pro-inflammatory M1 phenotype becomes dominant under pathological conditions, such as IBD. The M2 population, which is responsible for tissue repair, is severely depleted in IBD (13). M1 macrophages secrete abundant pro-inflammatory cytokines, recruit neutrophils (37), and drive the aberrant activation of T helper 17 (Th17) and T helper 1 (Th1) cells (38), thereby amplifying the immune response. However, the classical M1/M2 dichotomy represents an oversimplification of the actual complexity of intestinal macrophages in IBD. Recently, advances in single-cell and spatial transcriptomics have unveiled the higher-dimensional heterogeneity of macrophage transcriptional states. Single-cell RNA sequencing (scRNA-seq) of inflamed intestinal tissues in IBD has not only further subtyped the M0, M1, and M2 populations but also identified a novel subset termed inflammation-dependent alternative (IDA) macrophages (39). Pseudotime analysis revealed a continuous cellular differentiation trajectory among M0, M2, and an intermediate transitional state, known as M2.2. The transcriptional signature of IDA macrophages partly overlaps with those of M1 and M2 macrophages. CosMx spatial molecular imaging showed that macrophage diversity is closely linked to spatial localization within the intestinal wall (39). In the healthy colon, M0 and M2 macrophages are localized closest to the mucosal surface. In IBD, M1 macrophages are primarily concentrated in ulcerated regions. IDA macrophages are widely distributed throughout the inflamed lamina propria and granulomas. These findings indicate that in IBD, macrophages exist along a continuous spectrum of activation states, and their functional roles are shaped by local signals and spatial localization. In IBD, macrophages frequently exhibit impaired efferocytosis, leading to subsequent necrosis and the accumulation of apoptotic cells in injured tissues. This event causes DAMPs to be released in large amounts, thereby generating a vicious cycle that amplifies inflammation on its own (40). Crucially, when M1 polarization occurs, nuclear factor kappa B (NF-κB) signaling is hyperactivated. It transcriptionally enhances different components of the NOD-like receptor pyrin domain-containing 3 (NLRP3) inflammasome and pro-interleukin-1β (pro-IL-1β), thereby priming macrophages into a highly sensitized state for pyroptosis (41). Concurrently, the invasion of pathogenic microbes due to mucosal barrier disruption, coupled with the release of DAMPs due to defective efferocytosis, intensifies pyroptosis. These pathological changes together lay the foundation for macrophage pyroptosis.

## 3 Molecular mechanisms of pyroptosis

Pyroptosis represents a pro-inflammatory variant of regulated cell death. In the late 20th century, it was initially discovered in macrophages responding to pathogens, such as *Bacillus anthracis* lethal toxin, *Shigella*, and *Salmonella* (42–44). Due to early research limitations, it was mischaracterized as apoptosis until the term “pyroptosis” was formally coined in 2001 (45).

GSDMs serve as the ultimate downstream effectors of all pyroptotic pathways. Mammalian GSDMs comprise six members, including GSDMA-F. Among them, GSDMF cannot induce pyroptosis due to the absence of a pore-forming domain (46). Pyroptosis-competent GSDMs share conserved structural features, comprising an N-terminal pore-forming and a C-terminal autoinhibitory domain (47). At rest, the C-terminal domain represses the N-terminus via intramolecular interactions (48). Specific cleavage at the linker region by upstream caspases or granzymes removes the C-terminal domain, releasing the N-terminal domain, which possesses lipid-binding activity (49, 50). The N-terminal domain targets and binds to negatively charged phospholipids on the inner leaflet of the plasma membrane, where it oligomerizes to form pores with a diameter of 10–14 nm. This disrupts the plasma membrane, leading to intracellular osmotic imbalance, rapid cellular swelling, cellular rupture, and the secretion of pro-inflammatory cytokines, such as interleukin-18 (IL-18) and IL-1β (47).

**Figure 2:**
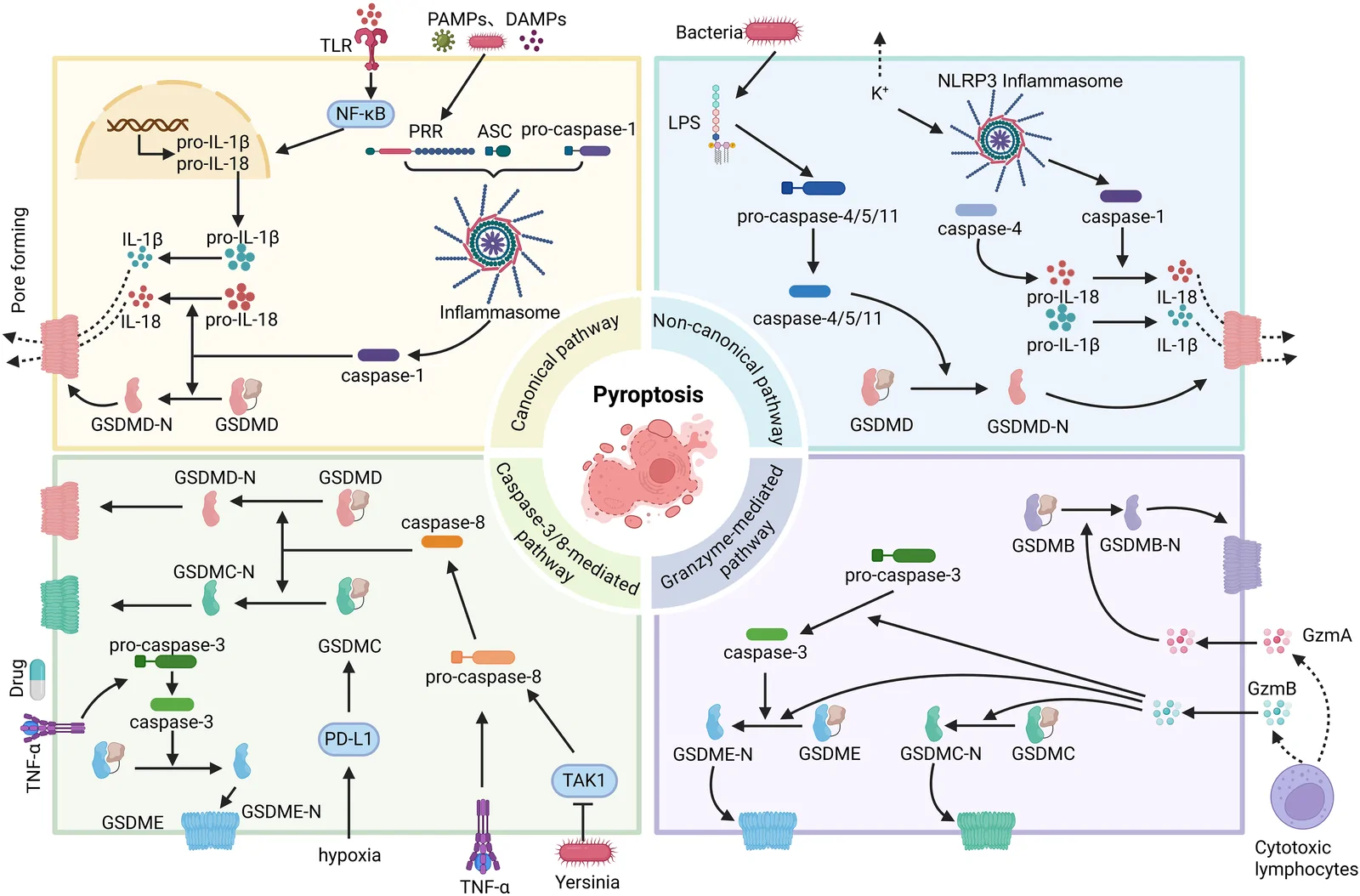
Molecular mechanisms of pyroptosis. Canonical pathway: The transcription of pro-IL-1β and pro-IL-18 is upregulated by the TLR/NF-κB signaling pathway; concurrently, PRRs recognize PAMPs or DAMPs to assemble the inflammasome and activate caspase-1. Active caspase-1 cleaves GSDMD to form membrane pores and processes pro-IL-1β and pro-IL-18 into their mature forms, which are subsequently released through these pores. Non-canonical pathway: Caspases-4/5/11 are directly activated by intracellular LPS to cleave GSDMD. Subsequent activation of the NLRP3 inflammasome and caspase-1 is triggered by GSDMD-mediated K^+^ efflux, which further promotes the maturation and release of IL-18 and IL-1β. Additionally, caspase-4 can directly process pro-IL-18. Caspase-3/8-mediated pathway: Without the involvement of inflammasomes, caspase-8 directly cleaves GSDMD and GSDMC, while caspase-3 directly cleaves GSDME. Granzyme-mediated pathway: GzmA directly cleaves GSDMB; GzmB directly cleaves GSDME and GSDMC, and can additionally activate caspase-3 to further process GSDME.

Under physiological conditions, moderate pyroptosis eliminates intracellular pathogens and modulates the immune system through the release of danger signals, thereby maintaining tissue homeostasis. Conversely, its aberrant activation triggers excessive inflammatory responses and culminates in tissue damage (51, 52). The aberrant activation of pyroptosis is a critical pathological mechanism involved in IBD (53). Thus, modulation of pyroptosis represents a highly promising therapeutic strategy (54). Based on distinct activation mechanisms, pyroptosis can be categorized into the canonical inflammasome pathway, the non-canonical inflammasome pathway, the caspases-3/8-mediated pathway, and the granzyme-mediated pathway (**Figure 2**).

### 3.1 Canonical inflammasome pathway

Two coordinated signals are required to initiate the canonical pathway (55). Toll-like receptors (TLRs) and other membrane-bound pattern recognition receptors (PRRs) provide the first signal. Pro-IL-1β and pro-IL-18 exhibit very limited basal expression levels in quiescent cells. TLRs and other membrane-bound PRRs are crucial for the transcriptional stimulation of pro-IL-1β and pro-IL-18. When TLRs identify PAMPs or DAMPs, they initiate downstream signaling to upregulate immature IL-1β and IL-18 (56). The second signal involves inflammasome assembly. The canonical inflammasome consists of three core components: intracellular sensor proteins, namely PRRs; the adapter protein, apoptosis-associated speck-like protein containing a CARD (ASC); and the effector protein, pro-caspase-1 (57). Under resting conditions, this complex is expressed at basal levels to maintain immune surveillance. Its activation in infection or tissue injury facilitates pathogen clearance and tissue repair. In contrast, excessive or aberrant activation of the inflammasome induces a profound inflammatory response (55).

The assembly and activation of inflammasomes rely on highly conserved domain interactions among their core components. The recognition of PAMPs and DAMPs by sensor proteins initiates this assembly process. Based on the specific sensor proteins, inflammasomes can be classified into families, such as the NOD-like receptor (NLR), AIM2-like receptor (ALR), and tripartite motif (TRIM) families, as well as other non-classical sensors (55). Using the NLR family as an archetype, these sensor proteins typically consist of three distinct domains: a C-terminal leucine-rich repeat (LRR) domain responsible for ligand recognition; a central nucleotide-binding (NACHT) domain that mediates oligomerization; and an N-terminal pyrin domain (PYD) or caspase recruitment domain (CARD) responsible for signal transduction (55, 57).

NLRs constitute the largest group of sensor proteins and are subclassified into NLRPs and NLRCs based on the presence of PYD and CARD in their N-terminus, respectively. Notably, NLRP3 can recognize a remarkably diverse array of stimuli. Since its N-terminus lacks a CARD, NLRP3 obligatorily relies on the adapter protein ASC to anchor pro-caspase-1 to assemble inflammasomes (58, 59). NLRP1 was the first identified inflammasome sensor (60). Its activation is uniquely mediated by functional degradation. The N-terminal fragment will be degraded following auto-cleavage within its FIIND domain, liberating the C-terminus to assemble the inflammasome (61, 62). Furthermore, human NLRP1 can directly recognize double-stranded RNA, which stimulates its ATPase activity and activates the inflammasome (63). In contrast, NLR family CARD domain-containing 4 (NLRC4) acts synergistically with NLR family apoptosis inhibitory protein (NAIP). Upon recognizing specific bacterial components, NAIP recruits and oligomerizes NLRC4, thereby promoting inflammasome assembly (64, 65). Notably, specific mutations in NLRC4 were shown to significantly increase susceptibility to UC (66, 67). The functions of other members of the NLR family have been elucidated in recent years. NLRP6 is highly expressed in the intestinal tract (68, 69), where it maintains intestinal homeostasis by combating viral infections and modulating the structure of gut microbiota (70–74). Furthermore, NLRP6 can sense endolysosomal damage to activate the inflammasome and induce pyroptosis (75). NLRP10 is an atypical sensor lacking the LRR domain (76). It can sense mitochondrial damage and assemble an inflammasome, thereby monitoring mitochondrial integrity in a mitochondrial DNA (mtDNA)-independent manner (77). NLRP10 protects the mucosal layer in the colon, and its deficiency was shown to exacerbate colitis (78). NLRP12 possesses dual pro-inflammatory and anti-inflammatory functions. It can form the NLRP12-PANoptosome complex to induce inflammatory cell death (79) and suppress excessive inflammatory responses through multiple mechanisms (80). Notably, genetic mutations in NLRP12 may be associated with the pathogenesis of CD (81, 82).

Beyond NLRs, absent in melanoma 2 (AIM2), which belongs to the ALR family, represents another classical sensor. It possesses a C-terminal HIN-200 domain. This domain specifically binds to double-stranded DNA (dsDNA). In its resting state, AIM2 adopts an autoinhibited conformation. The binding of dsDNA to the HIN-200 domain attenuates this inhibition and unmasks the N-terminal PYD. This conformational change facilitates the recruitment of the adaptor protein ASC. ASC is recruited via PYD-PYD homotypic interactions, which initiates downstream inflammasome assembly (83, 84). This DNA-sensing property is closely associated with the progression of IBD (85). In the dysregulated intestinal microenvironment, mucosal damage triggers the massive release of host-derived dsDNA. Gut microbiota dysbiosis also generates a high amount of exogenous dsDNA. Both dsDNA sources accumulate aberrantly. They induce a conformational change in AIM2, which triggers AIM2 activation, drives pyroptosis, and exacerbates intestinal injury (86, 87). Conversely, PYRIN, a member of the TRIM family, does not directly recognize pathogens. Instead, it indirectly detects bacterial toxins by monitoring the inactivation of host cell Rho GTPases (88). CARD8 is a human-specific sensor (89) that can directly recruit pro-caspase-1 to induce pyroptosis (90). Notably, mutations in CARD8 have been implicated in the pathogenesis of multiple autoimmune disorders, including IBD (91, 92).

ASC comprises a PYD and a CARD (93). The N-terminal CARD of pro-caspase-1 can bind to ASC or directly interact with CARD-containing sensors via homotypic CARD-CARD interactions. Upon the completion of inflammasome assembly, pro-caspase-1 undergoes auto-cleavage to generate catalytically active caspase-1. This active enzyme subsequently cleaves GSDMD, liberating its N-terminal domain (GSDMD-N), which possesses potent pore-forming activity on the cell membrane (47, 50). Caspase-1 acts concurrently. It converts the pro-IL-1β and pro-IL-18 into their mature forms. They are released into the extracellular space, recruiting immune cells and exacerbating the inflammatory response (57).

### 3.2 Non-canonical inflammasome pathway

In addition to the caspase-1-mediated canonical pathway, there exists a non-canonical inflammasome pathway mediated by caspases-4/5/11 (94). This pathway operates independently of canonical inflammasome sensors. Human caspases-4/5 and their murine ortholog caspase-11 recognize cytosolic lipopolysaccharide (LPS), cleaving GSDMD to execute pyroptosis. These caspases function dually as both PRRs and effector proteins. Bypassing the requirement of the adaptor protein ASC, they directly bind to invasive cytosolic LPS via their N-terminal CARDs (95, 96). Subsequently, activated caspases-4/5/11 directly cleave GSDMD (50, 97) and generate an N-terminal fragment that oligomerizes at the plasma membrane, leading to pore formation and driving pyroptosis-mediated cell lysis. This pathway plays a pivotal role in host defense against Gram-negative bacterial infections (94). Early studies suggested that caspases-4/5/11 cannot directly process pro-IL-1β or pro-IL-18; instead, their activity was thought to rely on the cross-activation of the canonical pathway. GSDMD-formed membrane pores trigger massive potassium (K^+^) efflux, which promotes NLRP3 inflammasome formation and indirectly activates caspase-1, leading to the maturation and secretion of IL-18 and IL-1β (94, 97). However, recent studies have reported that human caspase-4 can directly cleave pro-IL-18 and facilitate its maturation and release (98), utilizing the same cleavage site as caspase-1 (99). In contrast, the processing of pro-IL-1β still depends on indirect activation of the NLRP3/caspase-1 axis. Since Gram-negative bacterial infections are closely related to the pathogenesis of IBD, blocking this non-canonical pathway is a promising therapeutic strategy for treating IBD.

### 3.3 Caspases-3/8-mediated pathway

Pyroptosis and apoptosis were generally considered as two rather separate pathways in early studies. However, later studies have revealed that caspases-3/8, the classical caspases in apoptosis, are also intimately involved in pyroptosis. In cells expressing high levels of GSDME, caspase-3 cleaves GSDME specifically to release its N-terminal domain. It forms membrane pores and induces pyroptotic death (100). Initially, this mechanism was extensively investigated in the context of cancer immunology and targeted therapy (101). Furthermore, caspase-8 has been shown to cleave GSDMC or GSDMD. For instance, *Yersinia* infection was found to activate caspase-8 by inhibiting transforming growth factor-β-activated kinase 1 (TAK1), leading to GSDMD cleavage (102). Under hypoxic conditions, the nuclear translocation of PD-L1 upregulates GSDMC expression. Subsequently, tumor necrosis factor-alpha (TNF-α) activates caspase-8 to cleave GSDMC, inducing cancer cell pyroptosis and promoting tumor necrosis (103). These inflammasome-independent pathways are also aberrantly activated in IBD. TNF-α can upregulate caspase-3 via the transcription factor IRF1, and activated caspase-3 cleaves GSDME to induce pyroptosis, resulting in intestinal mucosal damage (104). Moreover, in the diseased tissues of patients with IBD and XIAP deficiency, caspase-8 overactivity directly cleaves GSDMD, thereby triggering macrophage pyroptosis (105).

### 3.4 Granzyme-mediated pathway

Early studies showed that granzyme B (GrB) can indirectly cleave GSDME by activating caspase-3 (106). However, recent studies have observed that the site on GSDME that is cleaved by caspase-3 is also directly targeted by GrB. This targeting leads to pyroptosis in target cells (107, 108), a pathway with significant potential for targeted therapy in cancer (109). Furthermore, GrB has been shown to cleave GSDMC, thereby inducing pyroptosis (110). In addition to GrB, granzyme A (GrA) was also reported to directly cleave GSDMB (49). Human GrA forms a unique exosite through dimerization, which specifically binds to the auto-inhibitory domain of GSDMB and executes precise cleavage at the Lys244 site (111). The pathway is regulated by the immune microenvironment and post-transcriptional modifications. Interferon-gamma (IFN-γ) can upregulate GSDMB expression (49, 112), and alternative splicing isoforms of GSDMB determine the probability of pyroptosis (113–115). High concentrations of IFN-γ markedly upregulate GSDMB in intestinal epithelial cells from CD patients. Consequently, CD8+ T cells secrete large amounts of GrA to specifically cleave GSDMB in target cells, directly triggering pyroptosis of intestinal epithelial cells (116). Targeted inactivation of GrA in models of intestinal inflammation inhibits activation of GSDMB and suppresses pyroptosis and inflammation, indicating a novel therapeutic target for IBD (117).

### 3.5 Crosstalk between pyroptosis and other programmed cell death pathways

There is considerable crosstalk between pyroptosis and other forms of programmed cell death, such as apoptosis and necroptosis (118). Apoptosis is characterized by cell shrinkage, membrane blebbing, and apoptotic body formation. These apoptotic bodies are subsequently cleared via phagocytosis to maintain immune tolerance (119). GSDMD serves as a critical intersecting node between pyroptosis and apoptosis. Caspase-1/4/5/11 cleave GSDMD to execute pyroptosis. Conversely, apoptotic caspases, such as caspase-3/7, can cleave GSDMD at distinct sites, inactivating the pore-forming activity of GSDMD and shifting cell fate toward apoptosis (120). Furthermore, caspase-3 can also cleave GSDME to trigger pyroptosis. This indicates that the same apoptotic executioner inhibits or promotes pyroptosis based on GSDM bioavailability (100). The NLRP3 inflammasome also exhibits functional plasticity. When the caspase-1 activation signal is compromised or delayed, the NLRP3-ASC complex can alternatively recruit caspase-8. This leads to the activation of apoptotic caspases rather than pyroptotic caspases (121). Necroptosis is mediated via the RIPK1-RIPK3-MLKL signaling axis. It is characterized by cell swelling and membrane rupture (122). Caspase-8 is a crucial molecular switch among these three pathways. When active, it inhibits necroptosis and promotes apoptosis. Under specific conditions, it can induce pyroptosis. On the contrary, caspase-8 inhibition contributes to RIPK1- and RIPK3-mediated necroptosis (123). Notably, pyroptosis, apoptosis, and necroptosis can be simultaneously activated via a shared platform termed the PANoptosome, forming a unique and integrated mode of cell death known as PANoptosis (124). This form of cell death has been strongly implicated in IBD (125). Recently, a “master molecular timer” model has been proposed. This model suggests that protein synthesis, post-translational modifications, and degradation engage in dynamic competition. This competition establishes a kinetic balance, thereby controlling pyroptosis, apoptosis, necroptosis, and PANoptosis (126). Selective inhibition of a single pyroptotic pathway might unintentionally divert cells to other alternative modes of death. Therefore, a profound understanding of the crosstalk between different death modalities and their underlying molecular mechanisms is required.

## 4 Pathological mechanisms of macrophage pyroptosis driving IBD progression

Single-cell transcriptomic studies have convincingly demonstrated the key role of macrophage pyroptosis in IBD. In CD, macrophages, especially those polarized to the M1 pro-inflammatory phenotype, exhibit significantly increased expression of GSDMD and multiple caspases. This suggests a greater susceptibility to pyroptosis (127). Compared with M2 macrophages, M1 macrophages exhibit higher levels of NLRP3 inflammasome activation and pyroptosis (128). Inflammasomes such as NLRP3 are highly expressed by immature macrophages infiltrating the inflamed intestinal mucosa of patients with CD, and their abundance correlates positively with disease activity (129). Similarly, scRNA-seq in the colonic lamina propria of UC patients showed that macrophages were the most correlated with pyroptosis among all immune cell subsets, with the majority of pyroptosis-related genes being expressed (130). During IBD progression, macrophage pyroptosis is not a simple cell death process. The massive release of intracellular contents after pyroptosis activates other immune cells. Furthermore, these mediators directly damage the intestinal epithelial barrier and serve as crucial factors involved in IBD progression via multiple mechanisms.

### 4.1 Multifaceted factors driving macrophage pyroptosis

In the microenvironment of IBD, macrophages exhibit heightened susceptibility to pyroptosis. Therefore, various signals can activate this cascade.

#### 4.1.1 Exogenous triggers

Gut microbiota plays a crucial role in the maintenance of intestinal homeostasis, and gut dysbiosis constitutes one of the key pathological factors participating in the development of IBD. The expansion of pathogenic bacteria and the concurrent increase in their products markedly upregulate PAMPs in the intestine, serving as vital exogenous signals that trigger macrophage pyroptosis. *Peptostreptococcus anaerobius*, a recognized pathogen, is significantly enriched in the gastrointestinal tract of patients with IBD, where it modulates the immune response and promotes colorectal carcinogenesis (131). Aberrant proliferation of *P. anaerobius* in the gut can activate TLR2/4, activating the NF-κB-NLRP3 signaling cascade to promote inflammasome assembly and macrophage pyroptosis, thereby exacerbating intestinal tissue damage (132). Furthermore, it has been demonstrated that *Escherichia coli* with high-pathogenicity islands directly enhances GSDMD-dependent canonical pyroptosis in macrophages (133). In addition to stimulation with intact whole bacteria, specific secretory products of pathogens can also induce macrophage pyroptosis. As a representative species of intestinal sulfate-reducing bacteria, *Desulfovibrio vulgaris* produces metabolites that disrupt the intestinal epithelial barrier (134, 135). In addition, flagellin released by *Desulfovibrio vulgaris* can activate the intracellular NAIP/NLRC4 inflammasome pathway, thereby inducing macrophage pyroptosis (136). Furthermore, outer membrane vesicles (OMVs) secreted by bacteria represent another critical pathogenic component capable of inducing macrophage pyroptosis. OMVs are nanoscale vesicular structures naturally released by bacteria during growth. They encapsulate bacterial contents, including LPS, proteins, nucleic acids, and virulence factors (137). These vesicles can pass through the intestinal barrier to enter the lamina propria (138), where they will be phagocytosed by macrophages (139). Nie et al. reported that OMVs secreted by *Desulfovibrio fairfieldensis* will be engulfed by macrophages, thereby activating the canonical caspase-1/GSDMD pyroptosis pathway, inducing macrophage pyroptosis, and promoting intestinal inflammation (140).

In addition to bacteria and their structural components, viruses can also serve as exogenous signals and trigger macrophage pyroptosis. There is a strong correlation between Epstein-Barr virus (EBV) infection and disease activity in IBD patients (141, 142). By comparing the colonic mucosa of EBV-positive and EBV-negative patients with UC, Ma et al. revealed that the expression of pyroptosis markers is significantly upregulated in CD68^+^ macrophages within the intestinal tissues of EBV-positive individuals. Furthermore, the expression levels of pyroptosis markers were shown to be positively correlated with clinical disease activity (143). Mechanistically, EBV infection induces metabolic reprogramming in macrophages, aberrantly shifting cellular energy metabolism from oxidative phosphorylation to glycolysis. Notably, treatment with the glycolysis inhibitor 2-DG significantly blocked virus-induced macrophage pyroptosis (144).

#### 4.1.2 Endogenous triggers

Beyond stimulation with PAMPs, endogenous signals, including DAMPs released from the damaged intestinal mucosa and the dysregulated expression of intrinsic molecules within host macrophages, can trigger macrophage pyroptosis. Mitochondrial dysfunction serves as a pivotal endogenous factor driving macrophage pyroptosis and IBD progression. Studies have shown that the GSDMD protein can undergo oxidative alteration due to mitochondrial reactive oxygen species (mtROS). This modification significantly enhances the cleavage efficiency of caspase-1, thereby facilitating NLRP3 inflammasome-dependent macrophage pyroptosis (145). The 18-kDa translocator protein (TSPO) is upregulated under normal inflammatory stress. It interacts with mitochondrial GSDMD to maintain the integrity of the outer mitochondrial membrane and inhibits excessive pyroptosis (146). However, this protective mechanism is abrogated in macrophages with TSPO deficiency. The resulting impairment in mitochondrial function leads to mtROS accumulation, which triggers the hyperactivation of NLRP3 and caspase-1 (147). These alterations ultimately induce GSDMD-mediated macrophage pyroptosis (148). Beyond mitochondrial dysfunction, aberrations in other organelles and their associated proteins are critical in pyroptosis (149). Patients with UC and murine models of colitis caused by dextran sulfate sodium (DSS) were reported to have considerably higher levels of pericentriolar material 1 (PCM1), a key component of centriolar satellites (150). Furthermore, PCM1 expression was positively correlated with inflammatory indices. In the intestine, aberrantly high expression levels of PCM1 exert a potent pro-inflammatory effect. By activating the NLRP3 inflammasome, PCM1 triggers GSDMD-dependent macrophage pyroptosis. Conversely, targeted knockdown of PCM1 effectively blocked the NLRP3/GSDMD pyroptotic axis, thereby alleviating intestinal mucosal injury (151).

Beyond intracellular organelles, specific receptors on the macrophage surface can also sense aberrant signals to promote pyroptosis. C-type lectin-like receptors serve as key molecules linking gut microbiota, epithelial barrier, and the immune system, playing a central role in the pathogenesis of IBD (152). Macrophage-inducible C-type lectin (Mincle), a PPR, not only recognizes exogenous pathogens but also acutely senses endogenous DAMPs (153). Within the complex pathological microenvironment of IBD, activation of the Mincle receptor rapidly triggers downstream pro-inflammatory signaling pathways. This not only elicits a robust inflammatory response but also maintains macrophages in a prolonged, hyper-sensitized pro-inflammatory state, thereby exacerbating the progression of colitis (154). Evidence from both patients with CD and murine colitis models indicates a significant upregulation of Mincle expression on the surface of intestinal macrophages. Upon the recognition of danger signals, Mincle rapidly recruits and activates the downstream spleen tyrosine kinase (Syk). Hyperactivation of the Mincle/Syk signaling pathway acts as a crucial factor driving the NLRP3 inflammasome expression, thereby promoting caspase-1 activation, GSDMD cleavage, and macrophage pyroptosis (155). V-set and immunoglobulin domain-containing 4 (VSIG4) is a negative immune-regulatory molecule specifically expressed on tissue-resident macrophages. VSIG4 expression was found to be inversely correlated with the activation level of the NLRP3 inflammasome. Its overexpression effectively suppressed NLRP3 inflammasome-mediated pyroptosis in macrophages and concurrently promoted macrophage polarization toward the M2 phenotype. These findings suggest that VSIG4 serves as a critical endogenous inhibitory factor for controlling pyroptosis in intestinal macrophages, and its deficiency can profoundly exacerbate intestinal inflammation (128). In addition to the aforementioned canonical pathways, dysregulated expression of triggering receptor expressed on myeloid cells 1 (TREM1) activates non-canonical pyroptosis. Studies have reported that in patients with IBD who do not respond to anti-TNF-α monoclonal antibody therapy, TREM1 is significantly upregulated on the macrophage surface (156, 157). Upon stimulation with high concentrations of TNF-α, TREM1 activates Syk kinase via its adaptor protein DAP12, which subsequently phosphorylates the E3 ubiquitin ligase ITCH at the Tyr772 residue. This modification enhances the enzymatic activity of ITCH, mediating ubiquitin-dependent degradation of TAK1. In macrophages, the loss of TAK1 activates cell death signals, culminating in the overactivation of caspase-8 and GSDMD cleavage (158).

In addition to intracellular proteins and membrane receptors, soluble inflammatory mediators in the extracellular microenvironment are critical for macrophage pyroptosis. Chitinase-3-like protein 1 (CHI3L1) is a secreted pro-inflammatory protein that was found to be significantly elevated in UC patients (159). In UC, macrophages abundantly produce and secrete CHI3L1 into their surrounding microenvironment (160). Studies have indicated that CHI3L1 specifically activates the BCAT1/NF-κB signaling axis, thereby inducing NLRP3/GSDMD-mediated macrophage pyroptosis. Either targeted knockdown of CHI3L1 or pharmacological intervention utilizing specific small-molecule inhibitors was shown to effectively abrogate BCAT1/NF-κB signal transduction. This targeted blockade significantly curtailed excessive macrophage pyroptosis *in vitro* and *in vivo* and markedly ameliorated colonic inflammation in murine models (161).

Pyroptosis has been strongly linked to non-coding RNAs (ncRNAs) (162). Circular RNAs (circRNAs) are a class of ncRNAs. CircRNAs are highly stable because of their covalently closed-loop structures, making them excellent biomarkers for disease status (163). Previous research showed that *hsa_circRNA_103124* expression levels are significantly increased in peripheral blood mononuclear cells of patients with active CD. In addition, this upregulation was positively correlated with the CD activity index and TLR4 expression. Experimental evidence showed that *hsa_circRNA_103124* activates the TLR4/MyD88 signaling axis and subsequently promotes downstream NF-κB activity. NF-κB activation was also found to promote the expression of the NLRP3 inflammasome and pyroptosis-related proteins, such as GSDMD, leading to macrophage pyroptosis (164).

Taken together, diverse and intricate factors collaboratively drive the aberrant pyroptosis of macrophages, which constitutes the core pathological mechanism driving intestinal inflammation and mucosal injury in IBD.

### 4.2 Macrophage pyroptosis-mediated release of inflammatory mediators and immune network remodeling

Numerous inflammatory mediators are released following macrophage pyroptosis. As necessary molecules for cell-cell communication, they have various interactions with different immune cells in order to profoundly remodel the immune microenvironment in the intestinal tract. The cytokines that are most commonly produced by pyroptotic macrophages include IL-1β and IL-18. Within the intestinal mucosa of patients with IBD, macrophages are a major source of them. These cytokine levels strongly correlate with IBD disease activity (129, 165). Under physiological conditions, basal levels of IL-1β contribute to tissue repair and the maintenance of immune homeostasis (166, 167). However, in IBD, IL-1β, a core biomarker of pyroptosis, exerts potent pro-inflammatory effects and exacerbates intestinal inflammation. IL-1β promotes neutrophil infiltration into the colonic mucosa (168) and concurrently induces the release of neutrophil extracellular traps (NETs), thereby aggravating mucosal damage. The amount of neutrophils entering the intestinal mucosa was thus shown to be considerably decreased by blocking IL-1β signaling (169). Furthermore, IL-1β can synergize with other inflammatory cytokines to induce naïve CD4^+^ T cell development into the pathogenic Th17 subgroup (170). Additionally, IL-1β directly modulates the function of γδ T cells, serving as a crucial upstream signal for their polarization into the γδT17 subpopulation and enhancing IL-17 secretion (171). Analogous to IL-1β, IL-18 released by pyroptotic macrophages plays a pivotal immunomodulatory role in IBD. The pro-inflammatory signaling of IL-18 is initiated through its direct ligation with the IL-18 receptors expressed on the surface of mucosal natural killer cells and group 1 innate lymphoid cells. This engagement triggers their aberrant activation, profoundly amplifies their cytotoxicity, and drives the production of IFN-γ (172, 173). IL-18 also profoundly modulates adaptive immune responses. IL-18, acting in synergy with interleukin-12, induces differentiation of CD4^+^ T cells into the Th1 lineage with a dramatic increase in IFN-γ secretion (174). IL-18 also induces the expression of interleukin-13 in cooperation with interleukin-2. This in turn promotes the polarization of CD4^+^ T cells toward the T helper 2 cell (Th2) phenotype (175). In the context of IBD, the over-activation of Th2 cells contributes to the progression of the disease through the overproduction of interleukin-4 and interleukin-5 (176, 177).

Pyroptosis results in a massive release of intracellular contents. Such mechanical disruption causes the uncontrolled release of a variety of crucial intracellular components. Under physiological conditions, these molecules are strictly sequestered intracellularly to maintain cellular homeostasis. However, once they are released into the extracellular milieu, they immediately act as danger signals. Neighboring cells detect these signals via PRRs, which trigger downstream inflammatory signaling cascades. High mobility group box 1 (HMGB1), a nuclear protein, is massively extruded into the extracellular space during pyroptosis. Both serum and colonic tissue samples from patients with IBD exhibited significantly elevated levels of HMGB1 (178, 179). In the murine models of DSS-induced colitis, the release of HMGB1 markedly exacerbated intestinal inflammation, whereas targeted inhibition of HMGB1 release markedly ameliorated inflammatory damage (180). Extracellular HMGB1 orchestrates the inflammatory response in the intestine by modulating a diverse array of immune cells. Studies have indicated that inhibition of HMGB1 attenuates NET formation and promotes the polarization of macrophages toward the M2 phenotype. This suggests that HMGB1 drives colitis progression fundamentally by regulating NET extrusion and macrophage plasticity (181). Additionally, HMGB1 can cause dendritic cells to release interleukin-23, which in turn causes group 3 innate lymphoid cells to release interleukin-22 and IL-17, exacerbating intestinal inflammation (182). By amplifying Th17 and Th1/Tc1 cell responses, HMGB1 was also reported to exacerbate colitis in murine models. Conversely, ethyl pyruvate, an established inhibitor of HMGB1, was shown to effectively alleviate intestinal inflammation by specifically blocking this pathogenic axis (183).

### 4.3 Macrophage pyroptosis-driven intestinal tissue injury

Beyond reprogramming the immune network, macrophage pyroptosis directly impairs intestinal epithelial barrier integrity. Under physiological conditions, subepithelial macrophages use their dendrite-like and balloon-like projections to sample luminal antigens and perform macropinocytosis without disrupting the epithelial barrier. Concurrently, they maintain intestinal homeostasis by removing apoptotic epithelial cells and cellular debris via efferocytosis (184, 185). However, in the presence of massive macrophage pyroptosis, cellular swelling and terminal rupture obliterate these delicate structures and profoundly impair the efferocytic capacity. Consequently, apoptotic cells and translocated luminal bacteria cannot be removed in a timely manner, exacerbating intestinal damage. Simultaneously, the massive efflux of intracellular contents released from pyroptotic macrophages results in the disintegration of intestinal epithelial integrity, which accelerates the development of IBD.

The integrity of the intestinal epithelial barrier strictly relies on the proper intercellular localization and stable levels of TJ proteins, primarily ZO-1, Occludin, and members of the Claudin family. Pyroptotic macrophage-derived inflammatory mediators can significantly disrupt the synthesis as well as spatial distribution of TJ proteins, thereby severely compromising intestinal barrier function (186, 187). Furthermore, released DAMPs, such as HMGB1, intensify the extent of damage to the epithelial barrier. Conversely, targeted inhibition of HMGB1 significantly upregulates the protein expression of TJ (188). Beyond junctional disruption, the massive efflux of intracellular contents from pyroptotic macrophages directly drives the death of intestinal epithelial cells (IECs). The overwhelming release of inflammatory cytokines acts as a potent trigger for IEC demise (189). Specifically, HMGB1 can interact with TLR4 on IECs, triggering profound lipid peroxidation and activating ferroptosis in these epithelial cells (190). Additionally, high concentrations of extracellular ATP can activate the endogenous NLRP3/caspase-1/GSDMD axis in IECs and induce their pyroptosis. Strikingly, these new pyroptotic IECs release even more ATP into the surrounding microenvironment. It generates a catastrophic amplification loop that accelerates mucosal destruction (191). Goblet cells, distributed among IECs, secrete mucins, predominantly MUC2. MUC2 is intricately linked to the formation of the protective mucosal layer. Pro-inflammatory cytokines are inversely correlated with MUC2 expression (192). Similarly, high levels of IL-18 compromise goblet cell functionality and suppress mucus production. Notably, in murine models of acute colitis, a monoclonal antibody specifically recognizing the neo-epitope of mature IL-18 rescued goblet cell function (193). In case of intestinal epithelial barrier dysfunction, luminal microorganisms and their derivatives translocate across the compromised barrier into the lamina propria. This overwhelming influx of PAMPs rapidly outstrips the clearance capacity of resident macrophages. Together with their already impaired efferocytic function, these persistent PAMPs trigger the pyroptosis of more macrophages, thereby perpetuating a catastrophic vicious cycle.

**Figure 3:**
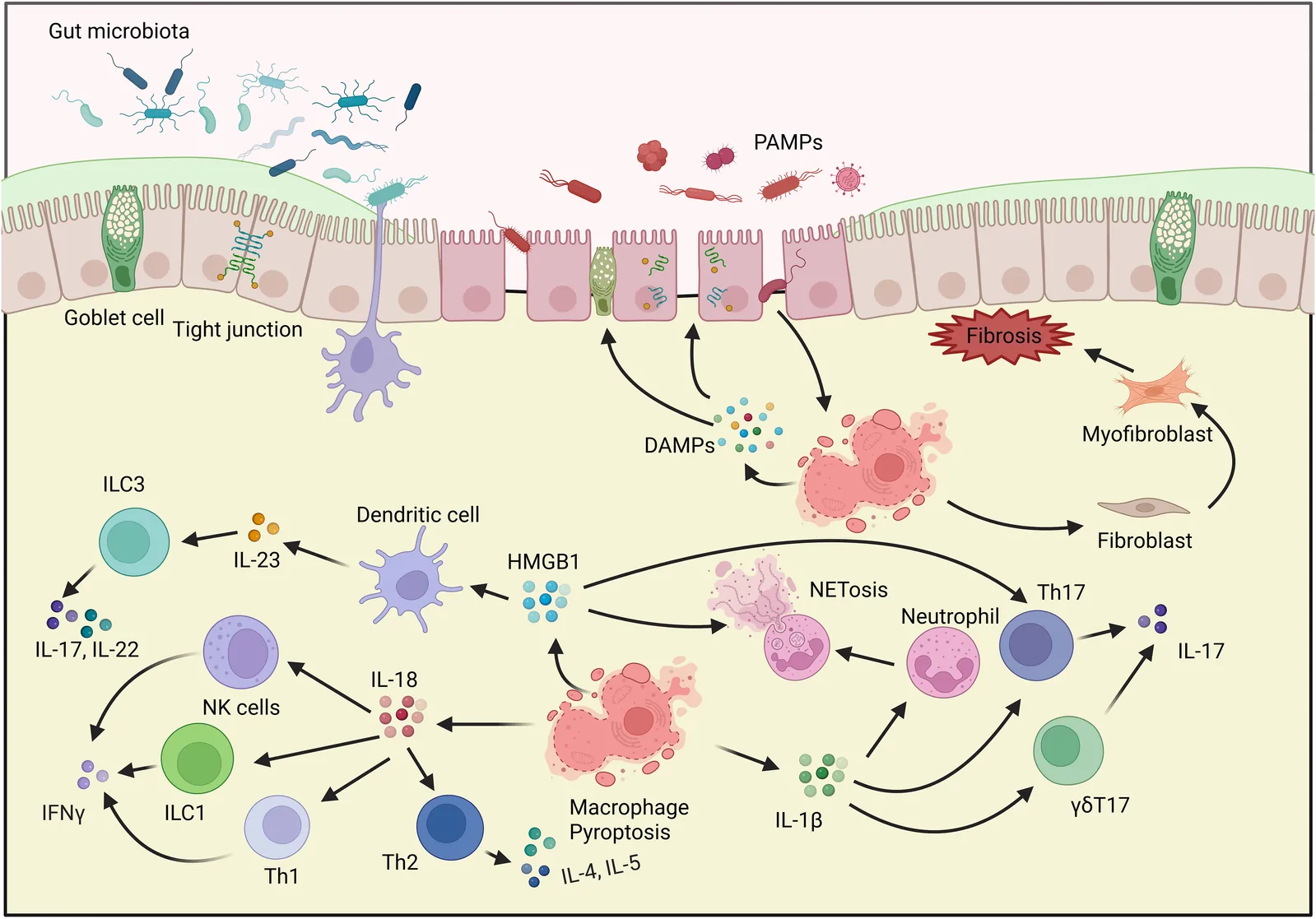
Macrophage pyroptosis drives immune network remodeling and intestinal mucosal barrier disruption in IBD. During macrophage pyroptosis, cell membrane rupture releases various mediators that remodel the immune network and disrupt tissue barriers. IL-1β promotes neutrophil recruitment and NET formation, and drives Th17 and γδT17 cells to secrete IL-17. IL-18 hyperactivates NK cells and ILC1s to secrete IFN-γ, while promoting the differentiation of Th1 and Th2 cells. Intracellular contents, such as HMGB1, promote NET formation and Th17 differentiation, and activate dendritic cells to secrete IL-23, which stimulates ILC3s to produce IL-22 and IL-17. Intestinal epithelial barrier is seriously compromised by mediators generated via macrophage pyroptosis, which also directly harm intestinal epithelial cells and affect tight junctions and goblet cell function. Concurrently, the loss of macrophage efferocytotic function leads to a massive influx of luminal pathogens and PAMPs, which triggers further macrophage pyroptosis and forms a vicious cycle. Chronic epithelial damage and recurrent inflammation drive fibroblast activation, leading to irreversible intestinal fibrosis.

The pathological consequences of macrophage pyroptosis extend far beyond acute inflammation and mucosal injury. In patients with CD, recurrent inflammatory episodes and persistent epithelial damage can induce intestinal wall fibrosis (3). Macrophage pyroptosis acts as a pivotal driver of this fibrotic cascade. The strictured intestinal tissues of CD patients have significantly higher numbers of macrophages that co-express GSDMD-NT and CD11b. GSDMD-knockout mice exhibited significantly milder intestinal fibrosis in TNBS-induced CD models. These findings compellingly suggest that targeted inhibition of AIM2-mediated pyroptosis in macrophages can significantly alleviate intestinal fibrosis in CD (194) (**Figure 3**).

## 5 Modulation of macrophage pyroptosis: a therapeutic approach to IBD

Currently, a wide variety of therapeutic agents are available for the clinical management of IBD. 5-Aminosalicylic acid (5-ASA) compounds are commonly used for the treatment of mild-to-moderate UC. Glucocorticoids are primarily employed for the induction of remission in acute, moderate-to-severe IBD. Conventional immunosuppressants, such as azathioprine and methotrexate, are mainly administered to maintain remission and reduce corticosteroid dependence. Biologics and small-molecule drugs, including anti-TNF-α inhibitors and S1P receptor modulators, can alleviate IBD by targeting specific inflammatory pathways (195). Among these agents, some were found to be associated with macrophage pyroptosis. For example, glucocorticoids were shown to suppress NLRP3 inflammasome activation and subsequent pyroptosis in macrophages through metabolic reprogramming (196). Studies have demonstrated that TREM1 diminishes the efficacy of infliximab by promoting caspase-8-dependent macrophage pyroptosis. This finding indicates that the therapeutic response to anti-TNF-α monoclonal antibodies is closely linked to macrophage pyroptosis (158). In addition, 5-ASA has been reported to modulate pyroptosis-related gene expression in IBD, and single-cell sequencing identified macrophages as the immune cell type most strongly correlated with pyroptosis (130). However, modulation of macrophage pyroptosis using 5-ASA warrants further investigation. Nevertheless, modulation of macrophage pyroptosis by available therapeutic agents is largely indirect, making precise and efficient blockade challenging. As the role of macrophage pyroptosis in the pathogenesis and progression of IBD becomes increasingly recognized, it is urgently needed to develop interventions specifically targeting macrophage pyroptosis. In recent years, various emerging approaches, including chemically synthesized inhibitors, biological agents, nanodelivery systems, and natural products and microbial metabolites, have shown potential in modulating macrophage pyroptosis.

### 5.1 Chemically synthesized inhibitors

Owing to their well-defined chemical structures and precisely elucidated mechanisms of action, chemically synthesized inhibitors occupy a prominent position in IBD therapy via modulation of macrophage pyroptosis. The synthetic small-molecule inhibitor VX765 potently suppressed the enzymatic activity of caspase-1 (197). In the murine models of spontaneous chronic colitis, the oral administration of the NLRP3 inflammasome inhibitor MCC950 effectively inhibited inflammasome assembly in bone marrow-derived macrophages and lamina propria macrophages. This targeted inhibition markedly curtailed the generation of active caspase-1 and the downstream secretion of inflammatory cytokines, thereby ameliorating the symptoms of colitis (198). In addition to directly targeting executing enzymes in pyroptosis, modulation of the upstream signaling networks via specific receptors represents a highly sophisticated therapeutic strategy in IBD. WAY200070 is a highly selective estrogen receptor β agonist. It actively downregulated the macrophage-localized surface calcium-sensing receptors. This action prevented the aberrant intracellular accumulation of Ca²^+^, blocked the NLRP3 inflammasome assembly, and attenuated pyroptosis-driven inflammation (199). Furthermore, the paradigm of drug repurposing highlights the untapped potential of established clinical therapeutics. LCZ696 is a well-known medication for heart failure. It alleviated mitochondrial dysfunction in macrophages, thereby reducing the production of mtROS. Remarkably, this targeted mitochondrial protection concurrently inhibited both the NLRP3 and the caspase-11/GSDMD pathways, thereby robustly ameliorating experimental colitis (200).

### 5.2 Biologics

Biologics encompass proteins, antibodies, and microbes generated via modern bioengineering techniques. These agents have demonstrated high efficacy in treating IBD (201). Recent developments in engineering biologics to target macrophage pyroptosis have yielded notable progress, particularly in the fields of microbiome-based therapeutics and monoclonal antibodies. In the realm of microecological interventions, lactic acid-producing *Saccharomyces cerevisiae*, rationally designed via synthetic biology, represents a major breakthrough. Lactic acid generated by this engineered yeast is internalized by macrophages via monocarboxylate transporter 1. When caspase-1 and the NLRP3 inflammasome are both strongly inhibited, macrophage pyroptosis is successfully prevented. This suppression happens after the substance is internalized (202). Consistently, the culture supernatant of *Saccharomyces boulardii* significantly ameliorated colitis. Mechanistically, analysis indicated that the supernatant upregulates farnesoid X receptor expression in macrophages, thereby inhibiting NLRP3 inflammasome activation and pyroptosis (203). Monoclonal antibodies play a central role in biologic therapies due to their ability to disrupt pathogenic signaling pathways through targeted blockade of immune cell surface receptors. There is hyperactivation of the Mincle/Syk signaling pathway in intestinal mucosal macrophages in patients with CD. Crucially, the anti-Mincle antibody 6G5 precisely uncoupled the Mincle receptor on the macrophage surface from its downstream Syk kinase, thereby suppressing macrophage pyroptosis (155).

### 5.3 Nanodelivery systems

The *in vivo* application of certain natural products and small-molecule agents is limited by poor aqueous solubility, low bioavailability, and a lack of target specificity. Furthermore, since macrophages reside in the intestinal lamina propria, therapeutic agents must traverse the intestinal epithelial barrier to reach their target site. Nanodelivery systems enable precise targeted delivery, thereby providing a novel technological platform for interventions regulating macrophage pyroptosis. Exosomes, naturally occurring nanovesicles secreted by cells, offer excellent biocompatibility and low immunogenicity. Human umbilical cord mesenchymal stem cell-derived exosomes (hucMSC-Exs) were demonstrated to home to inflamed tissues, serving as effective drug delivery vectors in IBD management. Studies have revealed that hucMSC-Exs stably and highly express miR-378a-5p. This microRNA was observed to directly bind to the 3’ untranslated region (3’UTR) of *NLRP3* mRNA in macrophages, thereby repressing the expression of NLRP3. Consistently, hucMSC-Exs curtailed macrophage pyroptosis and ameliorated intestinal damage in mice with DSS-induced colitis (204). Concurrently, exosomal miR-203a-3p.2 was shown to specifically target caspase-11/4. This interaction inhibited the non-canonical pathway of pyroptosis and potently suppressed inflammation (205). This multi-target and multi-pathway delivery paradigm offers a highly efficacious and safer therapeutic strategy for IBD. Beyond natural exosomes, synthetic nanoparticles can achieve active targeted delivery through surface functionalization. Disulfiram can strongly block pore formation mediated by GSDMD. However, its direct application has been limited due to the lack of targeting specificity and nonspecific toxicity. To overcome this issue, researchers encapsulated disulfiram within lactoferrin-based nanoparticles. This approach achieved precise targeted delivery due to the high expression of lactoferrin receptors LRP-1 on the surface of activated macrophages. It effectively blocked GSDMD-mediated membrane permeabilization and IL-1β release, thereby significantly mitigating inflammatory damage. Importantly, it achieved this while bypassing off-target adverse effects (206). In summary, by surmounting physiological barriers and enabling cell-specific targeting, nanodelivery systems are emerging as a pivotal bridge facilitating the clinical translation of anti-pyroptotic therapeutics.

### 5.4 Natural products and microbial metabolites

Natural products and microbial metabolites occupy a pivotal position in the therapeutic armamentarium for IBD (207, 208). Many studies have reported their capacity to effectively ameliorate IBD fundamentally by modulating macrophage pyroptosis (194, 209, 210).

Certain natural products can directly interfere with the assembly of inflammasomes. For instance, the diterpenoid Munronoid I enhanced K48-linked ubiquitination and subsequent proteasome-driven clearance of NLRP3 protein in macrophages. This degradation process effectively inhibited NLRP3 inflammasome formation and the downstream processing of GSDMD, thereby blocking macrophage pyroptosis and alleviating DSS-induced colitis (209). Alternatively, atranorin, a secondary metabolite derived from lichens, directly bound to the ASC protein in macrophages and prevented its oligomerization and the subsequent formation of the NLRP3 inflammasome (211). Narirutin, a phenolic compound extracted from dried tangerine peel (*Chenpi*), possesses anti-inflammatory and antibacterial activities (212, 213). Narirutin exerted its downregulatory effect on NLRP3 and IL-1β through inhibition of multiple signaling cascades, including MAPK, NF-κB, and the PI3K/AKT axis. Concurrently, disruption of the NLRP3-ASC interaction by this compound inhibited inflammasome assembly and ASC oligomerization and reduced IL-1β release and macrophage pyroptosis, thereby effectively alleviating colitis (214). In addition, arteannuin B, a sesquiterpene lactone extracted from *Artemisia annua*, suppressed NLRP3 inflammasome assembly and macrophage pyroptosis by directly binding to Gly271 and Ser331 of the NLRP3 NACHT domain. It disrupted the NLRP3-NEK7 interaction and ASC oligomerization, thereby alleviating DSS-induced colitis in mice (215). Notably, such intervention strategies are not restricted solely to the NLRP3 inflammasome. Imperatorin is a coumarin derivative from Apiaceae plants with well-established anti-inflammatory, antioxidant, and immunomodulatory effects (216). It significantly attenuated TNBS-induced intestinal fibrosis in CD models. Mechanistically, it suppressed the AIM2 inflammasome-mediated pyroptosis in macrophages (194). Besides, certain natural compounds were shown to modulate upstream cell surface receptors and their associated molecular signaling networks. For instance, salidroside is renowned for its antioxidant and anti-hypoxic properties (217). It precisely targeted the extracellular domain of TREM1. This targeting blocked the NLRP3 inflammasome assembly, inhibited macrophage pyroptosis, and ameliorated DSS-induced colitis (218). Piceatannol is a naturally occurring Syk kinase inhibitor. It specifically blocked the Mincle/Syk signaling cascade. This blockade significantly inhibited the NLRP3 inflammasome, prevented GSDMD cleavage, suppressed macrophage pyroptosis, and alleviated colitis (155). Honokiol, a polyphenolic compound isolated from *Magnolia officinalis*, was shown to directly target PPAR-γ. By modulating the PPAR-γ-TLR4-NF-κB axis, it suppressed the inflammatory response and specifically attenuated the GSDMD-mediated non-canonical pyroptotic pathway. This finding suggests the strong potential of honokiol to ameliorate UC by regulating macrophage pyroptosis (219). Similarly, baicalin downregulated TUG1 expression and disrupted the TUG1-PTBP1 interaction. This targeted intervention inhibited NLRP3 inflammasome activation. It suppressed macrophage pyroptosis and effectively alleviated UC (220). Furthermore, certain agents were found to potently inhibit pyroptosis by improving cellular metabolism and maintaining intracellular homeostasis. Saikosaponin A is the principal active saponin derived from the traditional Chinese medicine *Radix Bupleuri*. It was shown to target cholesterol 25-hydroxylase, thereby promoting the production of 25-hydroxycholesterol. This specific metabolic change successfully prevented the formation of inflammasomes and eventually reduced macrophage pyroptosis (221). Similarly, the natural compound indole-3-carbinol activated the aryl hydrocarbon receptor in macrophages, which upregulates ornithine decarboxylase 1 and drives spermine synthesis. By blocking K^+^ efflux, spermine inhibits NLRP3 inflammasome assembly and abrogates GSDMD-driven macrophage pyroptosis (222). Furthermore, nordihydroguaiaretic acid, a naturally occurring polyphenol generated via the metabolism of intestinal *Lactobacillus* species, was observed to maintain intestinal homeostasis by downregulating NR4A1 expression in macrophages. Low NR4A1 expression blocks NLRP3 inflammasome assembly and subsequent GSDMD cleavage, thereby inhibiting pyroptosis (223). Sodium butyrate (NaB) is a prototypic short-chain fatty acid generated by gut microbiota during the fermentation of dietary fibers. This microbial metabolite possesses profound anti-pyroptotic properties. NaB potently suppressed mtROS generation and preserved mitochondrial membrane potential. This crucial mitochondrial protection significantly limited NLRP3 inflammasome assembly and GSDMD cleavage. It effectively blocked macrophage pyroptosis and alleviated DSS-induced colitis (210).

Collectively, various natural compounds and microorganism-derived metabolites regulate macrophage pyroptosis via multi-target and diverse mechanistic pathways. A deeper understanding of their roles would help enhance the therapeutic options available to manage IBD.

The aforementioned intervention strategies have shown significant efficacy in relieving intestinal inflammation in IBD. However, their clinical translation warrants extreme caution. Importantly, pyroptosis has an intrinsic role in host defense and the maintenance of tissue homeostasis under physiological conditions. Nonspecific blockade of pyroptotic pathways may impair their inhibitory effects on other inflammatory pathways, which may paradoxically aggravate intestinal inflammation (224). Therefore, the key to future drug development is to achieve a fine balance between inhibiting pathological pyroptosis and preserving physiological immune defense (**Table 1**).

**Table 1:** Therapeutic interventions targeting macrophage pyroptosis in inflammatory bowel disease.

Category	Substance	Core targets	Mechanism of action	Models	Reference
Biologics	Lactic acid-producing *Saccharomyces cerevisiae*	MCT1; NLRP3 inflammasome; caspase-1	Generates lactic acid internalized via MCT1; suppresses NLRP3 inflammasome and caspase-1 activation	DSS-induced colitis in mice; LPS/ATP-stimulated BMDMs	(202)
	Saccharomyces boulardii	FXR; NLRP3 inflammasome	Activates FXR; suppresses NLRP3 inflammasome and caspase-1 activation	DSS-induced colitis mice; LPS/nigericin-stimulated THP-1	(203)
	Anti-Mincle neutralizing antibody 6G5	Mincle	Targets macrophage Mincle receptor; uncouples downstream Syk activation; suppresses NLRP3 inflammasome and caspase-1	DSS-induced colitis mice	(155)
Nanodelivery Systems	hucMSC-Exs (carrying miR-378a-5p)	miR-378a-5p; NLRP3 mRNA 3’UTR	Internalized by macrophages; binds to NLRP3 mRNA 3’UTR to suppress its expression; inhibits caspase-1 and GSDMD cleavage	DSS-induced colitis mice; LPS/nigericin-stimulated THP-1 cells and MPMs	(204)
	hucMSC-Exs (carrying miR-203a-3p.2)	miR-203a-3p.2; Caspase-11/4 mRNA 3’UTR	Internalized by macrophages; binds to the 3’UTR of caspase-11/4 mRNA to suppress its expression; blocks intracellular LPS-induced non-canonical pyroptosis and GSDMD cleavage	DSS-induced colitis mice; intracellular LPS-transfected THP-1 cells and BMDMs	(205)
	Disulfiram-loaded lactoferrin nanoparticles	LRP-1; GSDMD	Lactoferrin targets LRP-1 receptor on macrophages for active delivery; disulfiram inhibits GSDMD pore formation, blocking macrophage pyroptosis and IL-1β release.	DSS-induced colitis mice; LPS/nigericin -stimulated BMDMs	(206)
Natural Products and Microbial Metabolites	Munronoid I	NLRP3	Promotes K48-linked ubiquitination and proteasomal degradation of NLRP3; inhibits NLRP3 inflammasome activation and pyroptosis	DSS-induced colitis mice; LPS/ATP-stimulated MPMs	(209)
	Atranorin	ASC	Directly binds to ASC; restrains ASC oligomerization; inhibits NLRP3 inflammasome activation and cell pyroptosis	DSS-induced colitis mice; LPS/ATP-stimulated BMDMs	(211)
	Narirutin	NLRP3; ASC	Inhibits NF-κB, MAPK, and PI3K/AKT signaling to downregulate NLRP3 and IL-1β expression; blocks NLRP3-ASC interaction to inhibit inflammasome assembly and pyroptosis	DSS-induced colitis mice; LPS/ATP-stimulated BMDMs, THP-1	(214)
	Arteannuin B	NLRP3 (Gly271/Ser331); NLRP3-NEK7 interaction	Directly binds to Gly271 and Ser331 within the NLRP3 NACHT domain; sterically hinders NLRP3-NEK7 interaction; blocks ASC oligomerization and suppresses caspase-1 cleavage	DSS-induced colitis mice; LPS/ATP and other stimuli-stimulated BMDMs and THP-1	(215)
	Imperatorin	AIM2	Downregulates AIM2 expression; inhibits AIM2 inflammasome activation; suppresses caspase-1 and GSDMD cleavage	Stenotic ileocecal valve tissues from patients with CD; TNBS-induced intestinal fibrosis mice; LPS/poly(dA:dT)-stimulated J774A.1	(194)
	Salidroside	TREM1	Targets the extracellular domain of TREM1; blocks NLRP3 inflammasome assembly and GSDMD cleavage	DSS-induced colitis mice; LPS/ATP-stimulated BMDMs	(218)
	Piceatannol	Syk	Inhibits Mincle/Syk signaling; suppresses NLRP3 activation and GSDMD cleavage	DSS/TNBS-induced colitis mice; LPS + TDB-stimulated BMDMs	(155)
	Honokiol	PPAR-γ	Activates PPAR-γ; inhibits TLR4-NF-κB signaling; suppresses caspase-11/GSDMD-mediated non-canonical macrophage pyroptosis	DSS-induced colitis mice; RAW264.7	(219)
	Baicalin	TUG1/PTBP1/NLRP3 network	Downregulates TUG1 expression; disrupts TUG1-PTBP1 interaction; inhibits NLRP3 inflammasome activation	DSS-induced colitis mice; LPS/ATP-stimulated THP-1	(220)
	Saikosaponin A	CH25H/25-OHC axis	Directly binds to CH25H; promotes 25-OHC biosynthesis; inhibits NLRP3 inflammasome activation	DSS-induced colitis mice; LPS/ATP-stimulated BMDMs	(221)
	Indole-3-carbinol	AhR/ODC1/spermine axis	Activates AhR; promotes ODC1 transcription and spermine biosynthesis; suppresses K^+^ efflux; inhibits NLRP3 inflammasome assembly	DSS-induced colitis mice; LPS/nigericin -stimulated BMDMs	(222)
	Nordihydroguaiaretic acid	GSDMD/NR4A1/NLRP3 axis	Downregulates NR4A1 expression; disrupts NR4A1-NLRP3 interaction; inhibits NLRP3 inflammasome assembly and GSDMD cleavage	DSS-induced colitis mice; IL-10-deficient mice; LPS/ATP-stimulated BMDMs and RAW264.7	(223)
	Sodium butyrate	mtROS; NLRP3 inflammasome	Preserves mitochondrial integrity; inhibits mtROS production; suppresses NLRP3 inflammasome activation and GSDMD-mediated pyroptosis	DSS-induced colitis rats; LPS/Nigericin-stimulated RAW264.7	(210)

## 6 Conclusion and perspectives

Macrophage pyroptosis plays a pivotal role in the development and progression of IBD. Within the complex intestinal inflammatory microenvironment in IBD, macrophages undergo pyroptosis through multiple pathways. Pyroptotic macrophages and their products not only extensively reshape the intestinal immune microenvironment but also directly break the intestinal epithelial barrier, thereby contributing to the progression of IBD via several pathways. Currently, targeting macrophage pyroptosis has made substantial progress, demonstrating significant protective effects on the mucosal layer and alleviating disease severity in preclinical models.

Although targeting macrophage pyroptosis holds immense translational potential, its clinical application faces numerous challenges. Non-specific blockade of the pyroptotic pathways may paradoxically exacerbate intestinal inflammation by interfering with the physiological functions of pyroptosis. Previous studies on macrophage pyroptosis have predominantly relied on animal experiments and cell culture models, whereas clinical studies were frequently restricted by shortcomings such as small sample sizes and insufficient depth in terms of mechanistic exploration. Moreover, existing inhibitors of pyroptosis suffer from poor targeting specificity and low bioavailability. Due to the profound regulatory intersections between pyroptosis and alternative programmed cell death modalities, sole blockade of the pyroptotic pathway may disrupt the overall homeostasis of cell death and activate alternative death pathways.

Large-scale clinical cohort studies should be conducted in the future. By leveraging single-cell transcriptomics and spatial multi-omics technologies, researchers can precisely identify the specific subpopulations of macrophages driving pathological pyroptosis across different stages of IBD and explore their distinct molecular biomarkers. Furthermore, by integrating advanced targeted delivery systems and exploiting specific receptors on the surface of inflamed regions or activated immune cells, anti-pyroptotic drugs will more precisely target macrophages in damaged intestinal tissues. In-depth insight into how pyroptosis interacts with other forms of programmed cell death is critical for the successful management of IBD. In addition, novel inhibitors that have high selectivity and good bioavailability need to be developed. Structural biology can be used to achieve this goal. Through these concerted efforts, therapeutic strategies targeting macrophage pyroptosis can be successfully translated into clinical practice.
